# Ludwik Fleck’s analysis of public health nurses in the legitimization of the profession in Minas Gerais (1940–1970)

**DOI:** 10.1590/1980-220X-REEUSP-2024-0427en

**Published:** 2025-04-25

**Authors:** Bráulio Silva Chaves, Isabelle de Souza Januaria, Fernanda Batista Oliveira Santos

**Affiliations:** 1 Centro Federal de Educação Tecnológica de Minas, Departamento de Ciências Sociais e Filosofia, Belo Horizonte, MG, Brazil.; 2 Fiocruz Minas, Instituto René Rachou, Belo Horizonte, MG, Brazil.; 3 Universidade Federal de Minas Gerais, Escola de Enfermagem, Programa de Pós-Graduação em Enfermagem, Belo Horizonte, MG, Brazil.

**Keywords:** Life History Traits, History of Nursing, Public Health Nursing, Group Processes, Health Occupations

## Abstract

**Objective:**

To analyze the trajectory of three nurses – Rosa de Lima Moreira, Isaltina Goulart de Azevedo and Carmelita Pinto Rabelo –, considering their actions in Minas Gerais for the legitimization of nursing thought collective, based on Fleck’s ideas, between 1940 and 1970.

**Method:**

A qualitative and documentary study in the field of science history in interface with nursing history. Sources of different typologies and prosopographical nature were collected. The analysis considered Ludwik Fleck’s framework.

**Results:**

Historical findings indicate strong movement of these women in educational and associative institutional spaces in favor of public health in Minas Gerais, with agendas for training technical professional staff in health education at the Minas Gerais School of Public Health, in spaces within the scope of undergraduate and graduate studies at the *Escola de Enfermagem da Universidade Federal de Minas Gerais*, and militancy for the profession at the Brazilian Nursing Association.

**Conclusion:**

The intersection of trajectories shows that the triad of nurses from Minas Gerais sought legitimacy for nursing, contributing to a professional and epistemological inflection.

## INTRODUCTION

The serious public health situation in Brazil in the 1920s led Carlos Chagas, director of the Brazilian National Department of Public Health, to seek a nursing training model in the United States that could deal with the situation based on health education. The Brazilian National Department of Public Health School was then created in 1923 in the then federal capital, Rio de Janeiro, which gave rise to the current *Escola de Enfermagem Anna Nery* (EEAN) of *Universidade Federal do Rio de Janeiro* (UFRJ)^(1)^. In the wake of this professionalization movement on national soil, *Escola de Enfermagem Carlos Chagas* (EECC) was created in 1933, currently the *Escola de Enfermagem da Universidade Federal de Minas Gerais* (EEUFMG)^(2)^, where Rosa de Lima Moreira, Isaltina Goulart de Azevedo and Carmelita Pinto Rabelo studied and worked – the triad of nurses in this research.

The Brazilian social, political and economic context in the 20^th^ century was marked by democratic instability and moments of strong state authoritarianism. These aspects influenced the health sector organization, especially from a health perspective^(3)^. Thus, professionals focused on educating the people gained prominence, such as public health nurses and other technical stratifications, such as health visitors and educators and school health coordinators^(4)^. It is worth highlighting that, until the 1940s, the task of training health education professionals in Brazil was performed mainly by physicians. However, this scenario has been changing, with the influence of an international agenda promoted by the World Health Organization^(5)^.

The 1960s were particularly marked by the military (1964) at the center of political decisions. Regarding the health system, the country experienced a duality between social security assistance and public health. The former had actions aimed at formal workers’ individual health, and the latter, under the command of the Ministry of Health, was mainly aimed at rural areas and the poorest sectors of the population, and mainly targeted preventive activities^(6)^.

Although EEAN and EECC were created in this context of different public health demands in Brazil, the interests of nurse leaders of these institutions were focused not only on this field, but also on others^(7)^. In other words, it was important to legitimize the nursing profession in its various possibilities and different areas of activity, such as schools, health centers, hospitals, companies, etc.^(2)^.

In particular, EECC was an institution funded by the government of the state of Minas Gerais (MG)^(8)^. This situation differed from EEAN, which soon became federalized and became part of what would become UFRJ^(7)^. During its first 17 years of operation, EECC had many difficulties in graduating enough women to meet the public health demands of the state of MG. These difficulties can be explained by several factors, including: the status of women in Brazilian society at that time, with few opportunities for employment; the three-year course; and the low attractiveness of a nursing career in this training model^(8,9)^. Thus, EECC faced a serious crisis in the late 1940s, lacking the minimum conditions for its maintenance. The MG government gradually left the EECC aside, culminating in the creation *of Escola de Saúde Pública de Minas Gerais* (ESP-MG), which offered short courses (3 to 6 months), such as that for health educators, which pragmatically immediately met some demands for technical staff^(9)^.

In this context, three names in MG nursing that emerged between the 1940s and 1970s stand out: Rosa de Lima Moreira, Isaltina Goulart de Azevedo and Carmelita Pinto Rabelo. All three graduated in nursing from EEUFMG, were active in public health and held positions in the institution’s management, being recognized for their important role in nursing training and professionalization for the State and for Brazil^(10)^. The three also have a point of convergence in their trajectories, which was working in health education training courses at ESP-MG^(11)^. These points of agreement raised the following questions: who are these women who began to take on roles as professors and coordinators of health education courses, fostering the professionalization of nursing? Where did they come from, what was their training and their profile of work? To what extent is it possible, based on their trajectories, to establish a circulation network within public health structures, as well as other academic spaces, such as EEUFMG and ESP-MG? How do their professional lives help to build a part of nursing history in the period analyzed?

It is assumed that a look at the trajectory of these women contributed to a greater dimension in nursing history and its paths, through other variables, such as local, regional, contemplating the approaches with other collectives as defining scenarios of changes for health and for nursing itself.

In this regard, Ludwik Fleck, a Polish epistemologist, reflects on how a “collective of thought” is formed, based on a set of actions of subjects in various social spheres, contributing to thinking about this process for nursing. Fleck helps to problematize the movements within and outside nursing associated with the changes in health models that marked the post-Second World War period, reconfiguring nursing as a profession^(12)^.

These are new “styles of thought” that update nursing practices in light of the scenario of Brazilian instability and international health demands, in dialogue with regional aspects that mark the specificity of institutions. For these analyses, gaps were considered when it comes to a more comprehensive look at the importance of women in science and public health history in the state of MG.

Thus, the present study aimed to analyze the trajectory of three nurses - Rosa de Lima Moreira, Isaltina Goulart de Azevedo and Carmelita Pinto Rabelo -, considering their performances in MG for the legitimization of nursing thought collective, based on Fleck’s ideas, between 1940 and 1970.

## METHOD

### Study Design

This is research in sciences history in interface with nursing history^(13)^. As an approach, the COnsolidated criteria for REporting Qualitative research (COREQ), version in Portuguese spoken in Brazil^(14)^, were used, based on documentary sources and prosopography, starting from individual biographies to compose a social biography^(15)^. The analysis was supported by Ludwik Fleck’s epistemological thought framework^(12)^.

### Local

The study setting includes the locations where the triad of public healthcare professors worked in the 1940s and 1970s. This time frame encompasses the period in which courses on health education were offered at ESP-MG (the first in 1947 and the last in 1968 - the period of tropical medicine), and professors’ transition to EEUFMG. The two main locations are health education institutions in Belo Horizonte, MG, such as ESP-MG and EECC, which gave rise to EEUFMG.

### Selection Criteria

ESP-MG and the UFMG *Centro de Memória da Escola de Enfermagem* (CEMENF) have in their collection a documentary collection on education courses, which were chosen for this research.

Documentation was approached qualitatively, considering some inferences from the research: the importance of health education courses as spaces for expanding women’s performance and highlighting nurses working in public health; the movements undertaken by women in health institutions in MG and how they impacted consolidation of nursing in the period researched; the cut in the trajectory of three women who exemplify the role of public health nursing in training at various levels; and the approach to research and graduate studies in associations and management bodies.

Access to the sources followed the following stages: 1) collection of sources at ESP-MG on health education courses (health visitors and school health coordinator) between the 1940s and 1960s; 2) organization of documentation and its stratification, using data tabulation to compose the scenario; 3) selection of women who worked as professors and course coordinators; 4) cross-referencing with the CEMENF database, Carlos Chagas collection, seeking to determine which professors and coordinators had graduated in nursing at EEUFMG; 5) data collection on the transit of these women in institutions and in health management, based on the documentation found; 6) selection of three women, due to their importance in the nursing collective and in public health, considering the fact that they held teaching, management and even directorship positions at EEUFMG.

### Data Collection

At ESP-MG, data were collected from March to August 2021, and at CEMENF, from October 2023 to March 2024.

### Data Analysis and Treatment

The findings of the trajectories of the prosopographed social actresses were analyzed in the framework proposed by Fleck, in which the biographies are social subjects that reflect a “collective of thought” in nursing during the period studied and the correlation between them and the new “styles of thought” in health sciences^(12)^.

### Ethical Aspects

The study was conducted in accordance with national and international ethics guidelines. The documentary *corpus* was constituted by searches in two public collections, with no conflict of interest and waiving the need for the opinion of a Research Ethics Committee, as provided for in Resolutions 466/2012 and 510/2016 of the Brazilian National Health Council.

## RESULTS

### Three Women who Legitimize Nursing on Different Fronts and in Crossed Trajectories

Rosa de Lima Moreira was born on August 30, 1905, in the countryside of MG. She spent most of her life in the city of Belo Horizonte, the state capital, with her family, who owned a pharmacy in the Horto neighborhood. Under her father’s influence, at the age of 16, while single, she enrolled in the pharmacy course at the Belo Horizonte School of Dentistry and Pharmacy. In 1921, she graduated in pharmacy and inherited her father’s pharmacy. Still under her father’s influence, she enrolled in the second nursing class at EECC.

She graduated in 1936 and was invited to become an instructor at the institution. Between 1939 and 1948, she was part of school’s management as vice-principal and, between 1948 and 1950, as director of EECC. In 1950, no longer as director, Rosa de Lima witnessed the federalization process of EECC.

Later, she joined ESP-MG as a professor on the health visitor course (1953–1955), teaching topics in nursing history and ethics. In March 1954, Rosa was again invited to take over the directorship during the management of the Sisters of Charity. She remained in the position until March 1955.

In 1960 and 1961, she completed an internship in Bahia, receiving funding for a clinical medical internship at the *Universidade Federal da Bahia* School of Nursing. In October 1961, she visited EEAN to update her studies on the clinical medical scenario in the city of Rio de Janeiro. During this visit, Rosa de Lima was invited by EEAN director Waleska Paixão to take part in an extension course on the same topic.

In 1968, with the University Reform, conducted within the scope of EECC by professors Isaltina Goulart and Carmelita Rabelo, we witnessed the process of secularization and the reconfiguration of EECC in EEUFMG.

Rosa de Lima was always involved in nursing associations through the Brazilian Nursing Association, MG section (In Portuguese, *Associação Brasileira de Enfermagem, seção Minas Gerais* - ABEn-MG). She held positions on the fiscal council, such as from 06/23/1962 to 07/26/1964 and from 08/18/68 to 07/07/1970. She retired in 1970 from EEUFMG, and died on 05/08/2003.

**Figure 1:**
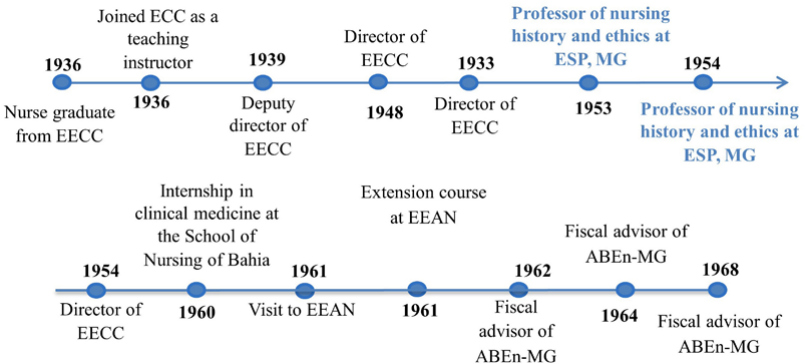
Rosa de Lima Moreira’s professional timeline.

Figure 1 highlights the main milestones in Rosa de Lima’s professional career.

Isaltina Goulart de Azevedo was born on March 19, 1917, in Itajubá, MG. While still in her hometown, she completed primary school (1931) and high school (1936). She worked as a professor for ten years, when in 1947 she joined EECC.

She was invited to teach at the institution in 1949, even before graduating, since there were no professors to teach “communicable diseases”. To take on this responsibility, the acting director of EECC, Rosa de Lima Moreira, sent Isaltina Goulart to Santos, São Paulo, to take a course with clinical practice/internship on this subject.

Having graduated as a nurse in 1950, she continued to work as a professor at EECC. She joined the Health and Welfare Department of the state of MG, with links to *Escola de Auxiliares de Enfermagem da Cruz Vermelha Brasileira-MG* (1951 to 1962) and to ESP-MG, in the health visitor course (1952 to 1956), teaching topics on nursing history, ethics, public health and communicable diseases.

Wanting to improve her teaching skills, she studied philosophy at UFMG, graduating in 1955. She had a very active professional life: she participated in conferences and held positions at ABEn. In 1962, she requested leave from her teaching activities to pursue a graduate course in nursing specializing in communicable diseases in the United States, with a scholarship funded by the Rockefeller Foundation.

Upon returning to Brazil in 1964, she expanded the scope of her partnerships, still with the support of the Rockefeller Foundation (a philanthropic institution that had been active in Brazil since the beginning of the 20^th^ century), with the Department of Preventive Medicine of the UFMG School of Medicine and with the World Health Organization, teaching and giving lectures on nurses’ work. In the same year, she took over as head of the MG Health Department Nursing Service (1964 to 1968). During this period, she organized the Infectious and Tropical Diseases Pavilion of the UFMG School of Medicine and took on the coordination of vaccination campaigns in the state of MG, as well as inspecting nursing assistant courses in the countryside of MG, due to her concern with internalizing good health practices.

She was appointed director of the School of Nursing in November 1968, for a three-year term, and was the first administrator of the institution to have a seat on the University Council. From 1950 to 1968, when EECC was attached to the UFMG School of Medicine, the nursing course director did not have administrative autonomy within the university, and had to direct all her demands to the director of medicine. Even at a time when EECC was subordinate, she served as a jury member for public examinations and awards, and was heavily involved in nursing congresses.

In July 1970, she took over the presidency of ABEn-MG, remaining until 1972. She maintained her articulation with classes and lectures on public health, ethics and nursing history between EE-UFMG/Faculty of Medicine of UFMG/*Hospital das Clínicas* of UFMG/dean’s office of UFMG/ESP-MG/Health Department of MG.

In 1971, she requested support to leave the country to study in England, as she had received an invitation from the British Council, and was supported with a scholarship from this entity. In 1972, she was also supported by the French embassy, at the request of UFMG’s dean office, to learn about nursing services in these countries. During the following year, 1973, Isaltina Goulart participated in the National Nursing Congress in Portugal and gave the opening lecture “The role of nurses in the modern world”, presenting overviews and perspectives of this professional in the countries she had visited.

Upon returning to Brazil, Isaltina Goulart began investing in research with the project “Study of the situation of *Hospital das Clínicas* of UFMG – as a training ground for nursing staff”. This was a major proposal to reorganize the hospital’s sectors to meet internships’ needs for EEUFMG. She assumed that the institution, despite being a teaching hospital, did not have the minimum structural conditions for nursing training. Thus, she conducted a situational diagnosis of these units, and the proposed improvements would be based on teaching hospitals in England, the birthplace of institutionalization of nursing education.

In 1981, Isaltina Goulart was appointed by the Departmental Council of UFMG School of Nursing to serve “*pro tempore*” as director of the aforementioned unit until the appointment of a new director.

Isaltina Goulart was the author of scientific texts, some examples of which include the text “Nursing and the nurse”, in which she exposes the lack of legitimacy in nursing and how this contributes to the spread of prejudice against this profession. Another text written by Isaltina at an event in Portugal was “The role of nurses in the modern world”, in which she once again evokes the importance of nursing for Brazilian health. She became professor emeritus in 1984. She died on January 15, 2009.

**Figure 2:**
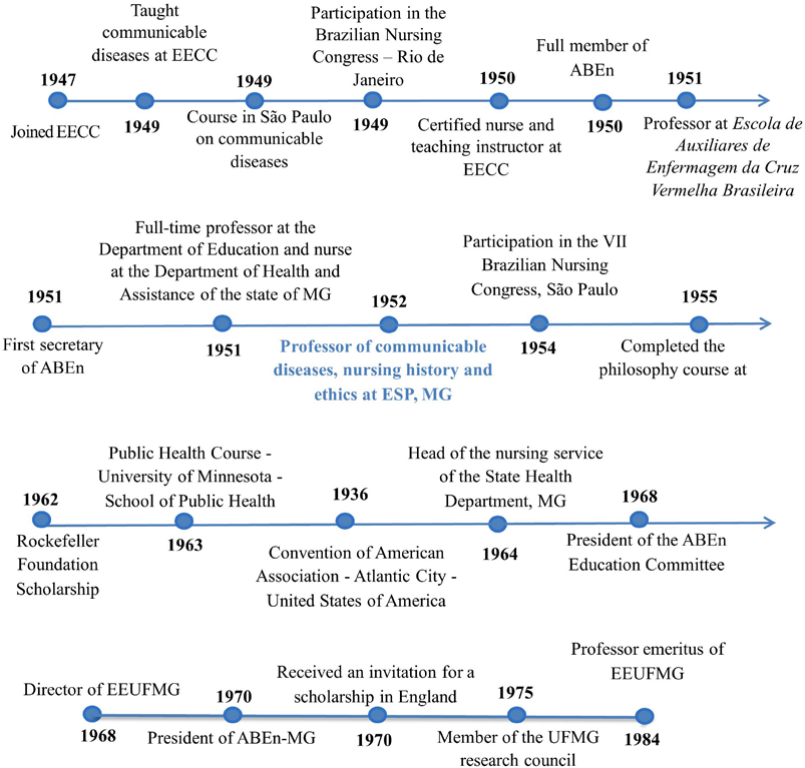
Isaltina Goulart’s professional timeline.

Figure 2 shows the main milestones in Isaltina Goulart’s professional career.

Carmelita Pinto Rabelo was born on September 16, 1937, in Bahia, where she completed her normal course at the age of 19. In 1957, she enrolled in the nursing course at EECC. She completed the course in January 1960 and, in February of the same year, became a professor at the institution.

Her first teaching experience was in public health, as it was the one that needed the most professors at EECC at that time. Also in 1960, she completed an internship in Pirapora, MG, in a mixed unit of the Special Public Healthcare service, a bilateral institution created with the so-called Washington Agreements (1942), with a strong presence in offering courses and assisting regional healthcare services, especially in the states of MG, Espírito Santo and Bahia. These experiences in public health led her to invest in a specialization course in public health at the School of Hygiene and Public Health at *Universidade de São Paulo* (USP) in 1961. In 1962, she worked as a professor in the health supervisor course at ESP-MG.

An activist for nursing, Carmelita Rabelo held positions at ABEn-MG, represented university collegiate bodies, headed the Department of Maternal and Child Nursing and Public Health at EEUFMG, participated in curricular reforms, and was involved in student activities and extension projects.

In 1967, she became the first lay director of EECC, after almost 20 years of religious leadership and administrative subordination to the UFMG School of Medicine. Her role as director allowed her to work outside the walls with UFMG dean’s office to separate the School of Nursing from the School of Medicine and to promote the institution’s autonomy after the 1968 Brazilian University Reform.

Later, she coordinated the first nursing entrance exam at UFMG, since admission until then was done through a simplified and non-unified exam process. From 1968 to 1972, she was vice-director of the School of Nursing, together with Professor Isaltina Goulart de Azevedo, when EECC was then called EEUFMG. A few years later, Carmelita completed her PhD degree in public health at the School of Public Health at USP in 1985. Carmelita Pinto Rabelo retired in 1991 and died on September 24, 2002.

**Figure 3:**
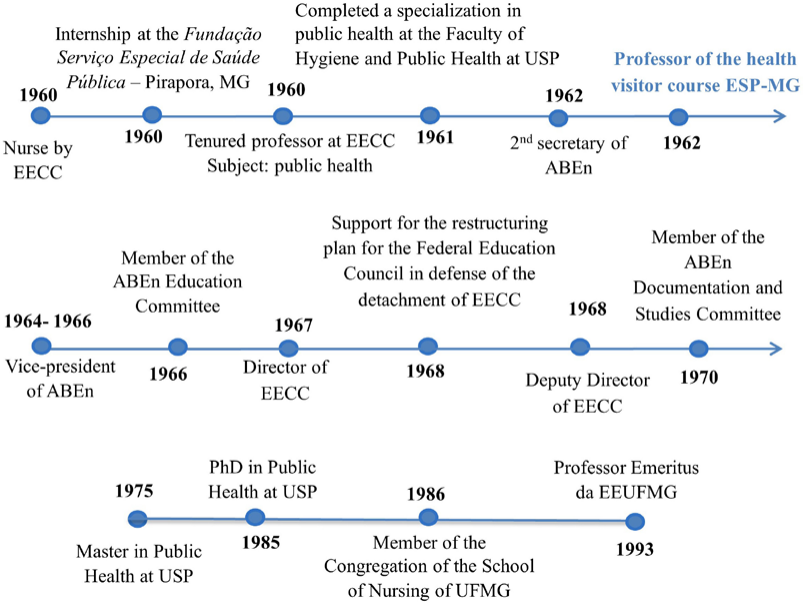
Carmelita Rabelo’s professional timeline.

Figure 3 below shows the main professional findings of Carmelita Rabelo’s career.

## DISCUSSION

Rosa de Lima Moreira’s, Isaltina Goulart de Azevedo’s and Carmelita Pinto Rabelo’s life trajectories have singularities in the feminine and professional universes as well as having differences of expression in the epistemic constructs of being/being/doing of nurses.

As for women, in the context of Victorian England, nursing became a career opportunity for white, single, high-society women. The institutionalization of knowledge was a differential to legitimize professionalization, as proposed by Florence Nightingale (1820–1910), recognized worldwide as the precursor of modern nursing^(16,17)^. Her model of teaching and training nurses was reproduced on all continents.

The impacts of institutionalization and internationalization achieved by the model proposed by Florence Nightingale arrived in Brazil at the beginning of the 20^th^ century, with the Carlos Chagas Reform, which incorporated it into the American version in 1922, which became known as the Anglo-American model. The materialization of this new “style of thinking” for nursing occurred with the creation of the Brazilian National Department of Public Health School of Nurses, which gave rise, in 1923, to the current EEAN of UFRJ, and, subsequently, to EECC, in 1933, the current EEUFMG^(18)^.

The triad of women who legitimized public health and nursing, described here, converged spatially at EECC in different historical periods: Rosa de Lima is among the pioneering students of EECC (1930s) as well as Isaltina Goulart (1940s) and Carmelita Rabelo (1950s). These periods reflect, respectively, some milestones: creation of the school under the tutelage of the MG state government; alignment with the Anna Nery standard – a model to be followed by Brazilian nursing schools created according to the norms of the Anglo-American system (1940s); and federalization of the institution to UFMG, incorporated and subordinated to the School of Medicine. Each of these women cements a stage of scientific and professional contribution to the consolidation of nursing, indicating a non-linear process with tensions unique to the times and contexts experienced by them and their category.

Despite the different historical times, the academic and professional socialization of these nurses took place in the same institution, with similar values and the search for an ethos for nursing. This certainly contributed to forging intellectual and ideological cohesion for common work fronts, such as the construction and expansion of their area of knowledge (nursing) in addition to their social responsibility for public health.

From an epistemological point of view, nursing is a practice that transforms itself based on changes in society^(19)^. As pointed out, this field puts pressure on the professionalization process, shaping new practices that will require adaptations to training and constant training so that nurses can meet formal and legal requirements, attentive to the historically established needs of their group and communities. As it produces knowledge, providing responses to the different health needs of the population, nursing is a science in which praxis and criticism must be part of the professional routine.

Whether due to social responsibility or for nursing in MG, a temporal and generational movement is observed in which one nurse prepares the field for another: a stage taken so that others can continue. In this way, articulated and collective movements are produced around nursing.

Rosa de Lima was a pharmacist and owner of a pharmacy, becoming a nursing student and professor in the 1930s. She assumed high-level leadership positions in the institution and at times of great vulnerability, such as when the director of the EECC (1940–1948): Waleska Paixão, left. Rosa de Lima was a character with an accurate vision of the demands of the time and of how nursing could contribute to a reciprocal movement to strengthen public health and establish its own professional field. Her continuity in these management positions allowed the EECC to exist and resist so that Isaltina Goulart, the second woman in the triad, could be a student and open the front for joint work with Carmelita Rabelo for secularization (the EECC was run by Sisters of Charity from 1950 to 1968). The end of the 1960s and beginning of the 1970s saw the Isaltina/Carmelita duo leading the first secular management of this institution after the University Reform of 1968, which incorporated, among other things, research as part of university life. From this fact onwards, both allowed the EECC, renamed EEUFMG, to have a seat on the University Council.

However, their actions and trajectories are not limited to EECC/EEUFMG. As women who are aware of the historical context, the three nurses occupied positions of power in different political and bureaucratic structures, such as ESP-MG (founded in 1946) and the State Department of MG, which were funded by agencies for professional training courses in other Brazilian states and abroad. These interactions demonstrate the scope of the expertise of these social agents in public health in MG, demonstrating how they expanded the status quo of nurses through their actions.

Another point to be observed is the triad’s adherence to associations in nursing. The prerogative of a strong profession after the implementation of the Anglo-American model in Brazil provided for the existence of an association so that nurses could bring together their interests. Thus, in 1936, the Brazilian National Association of Graduate Nurses was created, which gave rise to the current ABEn^(20)^.

Along with this, there is a concern to update nursing, beyond established practices, as a field of research. This is something that can be seen in Isaltina Goulart’s willingness and dedication to take the first stages towards the production of knowledge and research in nursing. Isaltina led the first research projects that considered nursing as the path to improving public health in the state^(21,22)^.

**Figure 4:**
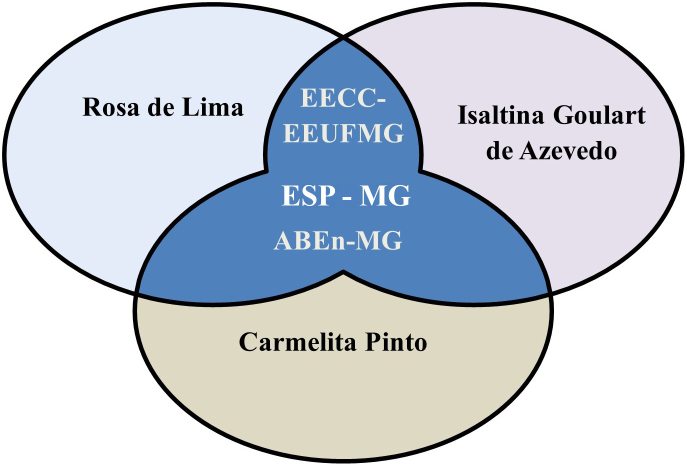
Consonance among social actors of tropical medicine.

By highlighting the trajectory of the triad of women who legitimized public health and nursing in MG, we see robust undertakings to raise nursing to new levels, larger and more in tune with the expectations of the university environment from the 1960s onwards. Thus, it is clear that each one, in their historical time, contributed in different ways to a larger process involving Brazilian nursing. By noting the path experienced by them, three emblematic spaces of health and nursing professionalization appear recurrently (Figure 4): EECC, ESP-MG, ABEn-MG.

The combination of prosopography with other methods can be used to broaden the understanding of social dynamics. Thus, this study presents unprecedented information about three women, nurses and pioneers in public health nursing. The intersection of their trajectories shows that the triad of nurses from MG sought legitimacy for nursing in the academic field and in associative life in favor of public health, contributing to a professional and epistemological inflection of the collective of thought in this field and in health.

The study limitations involved the lack of systematic information about certain moments in the careers of the social actresses mentioned, especially regarding their participation in ESP-MG. It is believed that failure to preserve sources from this documentary collection resulted in the loss of valuable information about their activities outside EECC/EEUFMG and ABEn circuits. However, assuming possible generalizations, the effort of these nurses to grow and value nursing in public health is highlighted.

## CONCLUSION

Rosa de Lima Moreira’s, Isaltina Goulart de Azevedo’s and Carmelita Pinto Rabelo’s careers help us understand that the process of inserting women into the field of public health in Brazil began more systematically in the post-World War II period, in the late 1940s. At that time, women entered the field at a very young age and even before finishing their regular studies. Another important element in the process of professionalizing the triad in the field of tropical medicine are public institutions and services such as ESP-MG and institutes. During their time at the different institutes, women “did it all”, occupying the most varied technical and intellectual production activities.
