# Supplementary material for: Ludwik Fleck’s analysis of public health nurses in the legitimization of the profession in Minas Gerais (1940–1970)
Source: Rev Esc Enferm USP. 2025 Apr 25;59:e20240427. doi: 10.1590/1980-220X-REEUSP-2024-0427en (PMC12024355; doi:10.1590/1980-220X-REEUSP-2024-0427en)
Supplement: Translation

# Translation: Análise fleckiana acerca das enfermeiras de saúde pública na legitimação da profissão em Minas Gerais (1940–1970)

**DOI:** 10.1590/1980-220X-REEUSP-2024-0427pt

**Authors:** Bráulio Silva Chaves, Isabelle de Souza Januaria, Fernanda Batista Oliveira Santos

**Affiliations:** 1 Centro Federal de Educação Tecnológica de Minas, Departamento de Ciências Sociais e Filosofia, Belo Horizonte, MG, Brasil.; 2 Fiocruz Minas, Instituto René Rachou, Belo Horizonte, MG, Brasil.; 3 Universidade Federal de Minas Gerais, Escola de Enfermagem, Programa de Pós-Graduação em Enfermagem, Belo Horizonte, MG, Brasil.

**Keywords:** Características de História de Vida, História da Enfermagem, Enfermagem em Saúde Pública, Processos Grupais, Ocupações em Saúde.

## Abstract

**Objetivo:**

Analisar a trajetória de três enfermeiras – Rosa de Lima Moreira, Isaltina Goulart de Azevedo e Carmelita Pinto Rabelo –, considerando suas atuações em Minas Gerais para a legitimação do coletivo de pensamento da enfermagem, a partir das ideias de Fleck, entre 1940 e 1970.

**Método:**

Estudo no campo da história da ciência em interface com a história da enfermagem qualitativo e documental. Foram coletadas fontes de tipologias diversas e de caráter prosopográfico. A análise considerou o referencial de Ludwik Fleck.

**Resultados:**

Os achados históricos indicam forte movimentação dessas mulheres em espaços institucionais educacionais e associativos em prol da saúde pública mineira, com agendas para formação de quadros profissionais técnicos na educação sanitária na Escola de Saúde Pública de Minas Gerais, em espaços no âmbito da graduação e pós-graduação na Escola de Enfermagem da Universidade Federal de Minas Gerais, e militância pela profissão na Associação Brasileira de Enfermagem.

**Conclusão:**

O cruzamento das trajetórias mostra que a tríade de enfermeiras mineiras buscou legitimidade para a enfermagem, contribuindo para uma inflexão profissional e epistemológica.

## INTRODUÇÃO

A conjuntura grave de saúde pública do Brasil nos anos 1920 fez com que Carlos Chagas, diretor do Departamento Nacional de Saúde Pública, buscasse nos Estados Unidos um modelo de formação em enfermagem que pudesse lidar com a situação, pautando-se na educação sanitária. Foi então criada, em 1923, na então capital federal, Rio de Janeiro, a Escola do Departamento Nacional de Saúde Pública, que deu origem à atual Escola de Enfermagem Anna Nery (EEAN) da Universidade Federal do Rio de Janeiro (UFRJ)^(1)^. Na esteira desse movimento de profissionalização em solo nacional, foi criada, em 1933, a Escola de Enfermagem Carlos Chagas (EECC), atual Escola de Enfermagem da Universidade Federal de Minas Gerais (EEUFMG)^(2)^, onde estudaram e atuaram Rosa de Lima Moreira, Isaltina Goulart de Azevedo e Carmelita Pinto Rabelo – a tríade de enfermeiras desta pesquisa.

O contexto social, político e econômico brasileiro no século XX foi marcado por instabilidades democráticas e por momentos de forte autoritarismo estatal. Tais aspectos influenciaram os caminhos de organização do setor saúde, principalmente do ponto de vista sanitário^(3)^. Assim, profissionais voltados para a educação do povo ganharam destaque, como a enfermeira de saúde pública e outras estratificações técnicas, como as visitadoras e educadoras sanitárias e as coordenadoras escolares de saúde^(4)^. Vale realçar que, até a década de 1940, a tarefa de formar os profissionais de educação sanitária no Brasil era desempenhada, sobretudo, por médicos. Porém, esse cenário foi se modificando, com a influência de uma agenda internacional propagada pela Organização Mundial da Saúde^(5)^.

A década de 1960 é particularmente marcada pelos militares (1964) no centro das decisões políticas. No que tange ao sistema de saúde, o país vivia a duplicidade entre a assistência previdenciária e a saúde pública. A primeira tinha ações dirigidas à saúde individual dos trabalhadores formais, e a segunda, sob o comando do Ministério da Saúde, era direcionada principalmente às zonas rurais e aos setores mais pobres da população, e tinha como alvo, majoritariamente, atividades de caráter preventivo^(6)^.

Apesar de a EEAN e a EECC terem sido criadas neste contexto de diferentes demandas de saúde pública do Brasil, os interesses das enfermeiras líderes dessas instituições estavam voltados não só para esse campo, mas também para outros^(7)^. Ou seja, interessava legitimar a profissão da enfermeira em suas diversas possibilidades e diferentes espaços de atuação, como as escolas, os centros de saúde, os hospitais, as empresas, *etc*.^(2)^.

Em especial, a EECC era uma instituição financiada pelo governo do estado de Minas Gerais (MG)^(8)^. Tal situação diferia da EEAN, que logo se federalizou e se tornou parte do que seria a UFRJ^(7)^. Ao longo dos seus 17 primeiros anos de funcionamento, a EECC apresentou muitas dificuldades em diplomar quantitativos suficientes para prover as demandas de saúde pública do estado de MG. Essas dificuldades podem ser explicadas por fatores diversos; entre eles, podemos destacar: a condição da mulher naquele momento na sociedade brasileira ainda com pouca saída para o mercado de trabalho; o tempo do curso com duração de três anos; e a pouca atratividade da carreira da enfermeira nesse modelo de formação^(8,9)^. Assim, a EECC enfrentou grave crise no fim dos anos 1940, faltando condições mínimas para a sua manutenção. O governo de MG foi progressivamente deixando de lado a EECC, culminando com a criação da Escola de Saúde Pública de Minas Gerais (ESP-MG), que formava com cursos rápidos (3 a 6 meses), como o de educadoras sanitárias, que pragmaticamente supriram de imediato algumas demandas de quadros técnicos^(9)^.

Nesse contexto, realçam-se três nomes da enfermagem mineira que despontam entre os anos 1940 e 1970: Rosa de Lima Moreira, Isaltina Goulart de Azevedo e Carmelita Pinto Rabelo. As três se formaram em enfermagem pela EEUFMG, eram atuantes na saúde pública e exerceram cargos na direção da instituição, sendo reconhecidas pelo seu papel importante na formação e profissionalização em enfermagem para o Estado e para o Brasil^(10)^. As três também possuem um ponto de convergência em suas trajetórias, que foi a atuação em cursos de formação em educação sanitária na ESP-MG^(11)^. Esses pontos de consonância suscitaram os seguintes questionamentos: quem são essas mulheres que passaram a assumir espaços como professoras, coordenadoras de cursos de educação sanitária, propiciando a profissionalização da enfermagem? De onde elas vinham, qual a sua formação e seu perfil de atuação? Em que medida é possível, a partir de suas trajetórias, estabelecer uma rede de circulação no âmbito das estruturas de saúde pública, além de outros espaços acadêmicos, como EEUFMG e ESP-MG? Como suas vidas profissionais ajudam a construir uma parte da história da enfermagem no período analisado?

Pressupõe-se que um olhar para a trajetória dessas mulheres contribuiu para uma dimensão maior na história da enfermagem e de seus percursos, por meio de outras variáveis, como locais, regionais, contemplando as aproximações com outros coletivos como definidoras de cenários de mudanças para a saúde e para a própria enfermagem.

Nesse sentido, Ludwik Fleck, epistemólogo polonês, reflete sobre como um "coletivo de pensamento" se forma, a partir de um conjunto de ações dos sujeitos em várias esferas sociais, contribuindo para pensar nesse processo para a enfermagem. Fleck ajuda a problematizar os movimentos dentro e fora da enfermagem associados às mudanças nos modelos de saúde que marcam o pós-Segunda Guerra Mundial, reconfigurando a enfermagem como profissão^(12)^.

Tratam-se de novos "estilos de pensamento" que atualizam as práticas da enfermagem diante do cenário de instabilidade brasileira e das demandas internacionais da saúde, em diálogo com aspectos regionais que marcam a especificidade das instituições. Para tais análises, consideraram-se as lacunas quando se trata de um olhar mais abrangente acerca da importância das mulheres na história da ciência e da saúde pública no estado de MG.

Desta forma, o presente estudo tem como objetivo analisar a trajetória de três enfermeiras – Rosa de Lima Moreira, Isaltina Goulart de Azevedo e Carmelita Pinto Rabelo -, considerando suas atuações em MG, para a legitimação do coletivo de pensamento da enfermagem, a partir das ideias de Fleck, entre 1940 e 1970.

## MÉTODO

### Desenho do Estudo

Trata-se de pesquisa do campo da história das ciências em interface com a história da enfermagem^(13)^. Como abordagem, utilizou-se s *COnsolidated criteria for REporting Qualitative research* (COREQ), versão em português falado no Brasil^(14)^, a partir de fontes documentais e prosopografia, partindo-se das biografias individuais para a composição de uma biografia social^(15)^. A análise teve sua sustentação no referencial do pensamento epistemológico de Ludwik Fleck^(12)^.

### Local

O cenário do estudo compreende os locais em que a tríade de professoras de saúde pública atuou nos anos 1940 e 1970. Este delineamento temporal compreende o período de oferta de cursos sobre a educação sanitária na ESP-MG (primeiro em 1947 e último em 1968 – período da medicina tropical), trânsito das docentes na EEUFMG. Os dois principais espaços são instituições de ensino de saúde em Belo Horizonte, MG, como ESP-MG e EECC, que deram origem à EEUFMG.

### Critérios de Seleção

A ESP-MG e o Centro de Memória da Escola de Enfermagem (CEMENF) da UFMG possuem em seu acervo uma coleção documental sobre os cursos de educação, sendo essas escolhidas para esta pesquisa.

A documentação foi abordada de forma qualitativa, considerando algumas inferências da pesquisa: a importância dos cursos de educação sanitária como espaços de ampliação da atuação das mulheres e de destaque para as enfermeiras com atuação na saúde pública; os movimentos empreendidos por mulheres em instituições de saúde de MG e como eles impactaram a consolidação da enfermagem no período pesquisado; o recorte na trajetória de três mulheres que exemplificam o papel da enfermagem de saúde pública na formação em diversos níveis; e a aproximação com a pesquisa e a pós-graduação no associativismo e nos organismos da gestão.

O acesso às fontes seguiu as seguintes etapas: 1) coleta de fontes na ESP-MG sobre os cursos de educação sanitária (visitadoras sanitárias e coordenadora escolar de saúde) entre as décadas de 1940 e 1960; 2) organização da documentação e sua estratificação, com o uso da tabulação de dados para composição do cenário; 3) recorte em mulheres que atuaram como professoras e coordenadoras dos cursos; 4) cruzamento com o banco de dados do CEMENF, coleção Carlos Chagas, buscando perceber quais as professoras e coordenadoras foram diplomadas em enfermagem na EEUFMG; 5) levantamento de dados sobre o trânsito dessas mulheres em instituições e na gestão em saúde, a partir da documentação encontrada; 6) recorte da pesquisa em três mulheres, pela sua importância no coletivo de enfermagem e na saúde pública, considerando o fato de terem exercido cargos de docência, gestão e, inclusive, direção na EEUFMG.

### Coleta de Dados

Na ESP-MG, os dados foram coletados de março a agosto de 2021, e no CEMENF, de outubro de 2023 a março de 2024.

### Análise e Tratamento dos Dados

Os achados das trajetórias das atrizes sociais prosopografadas foram analisados no arcabouço proposto por Fleck, em que as biografadas são sujeitos sociais que refletem um "coletivo de pensamento" da enfermagem no período estudado e a correlação entre elas e os novos "estilos de pensamento" das ciências da saúde^(12)^.

### Aspectos Éticos

O estudo foi conduzido de acordo com as diretrizes de ética nacionais e internacionais. O *corpus* documental foi constituído por buscas em dois acervos públicos, não havendo conflito de interesses e dispensando o parecer de Comitê de Ética em Pesquisa, conforme previsto nas Resoluções n^o^ 466/2012 e n^o^ 510/2016 do Conselho Nacional de Saúde.

## RESULTADOS

### Três Mulheres que Legitimam a Enfermagem em Diferentes Frentes e em Trajetórias Cruzadas

Rosa de Lima Moreira nasceu em 30 de agosto de 1905, no interior de MG. Residiu boa parte da vida na cidade de Belo Horizonte, capital do estado, junto à sua família, que possuía uma farmácia no bairro Horto. Sob influência do pai, aos 16 anos, solteira, ingressou no curso de farmácia na Escola de Odontologia e Farmácia de Belo Horizonte. Em 1921, diplomou-se farmacêutica e herdou a farmácia do pai. Ainda sob influência paterna, ingressou na segunda turma de enfermagem da EECC.

Formou-se em 1936, e foi convidada a ser instrutora da instituição. Entre 1939 e 1948, participou da direção da escola como vice-diretora e, entre 1948 e 1950, como diretora da EECC. Em 1950, não mais como diretora, Rosa de Lima presenciou o processo de federalização da EECC.

Posteriormente, vinculou-se como docente à ESP-MG no curso de visitadora sanitária (1953-1955), ministrando temáticas nas áreas da história da enfermagem e ética. Em março de 1954, Rosa foi novamente convidada a assumir a diretoria durante a gestão das irmãs de caridade. Ela se manteve no cargo até março de 1955.

Nos anos 1960 e 1961, realizou um estágio na Bahia, subsidiada com fomento para estágio de clínica médica na Escola de Enfermagem da Universidade Federal da Bahia. Em outubro de 1961, realizou uma visita à EEAN para atualizar estudos sobre o cenário da clínica médica na cidade do Rio de Janeiro. Nessa visita, Rosa de Lima foi convidada pela diretora da EEAN, Waleska Paixão, para fazer parte de um curso de extensão sobre a mesma temática.

Em 1968, com a Reforma Universitária, conduzida no âmbito da EECC pelas docentes Isaltina Goulart e Carmelita Rabelo, presenciou o processo de laicização e a reconfiguração da EECC em EEUFMG.

Rosa de Lima sempre esteve envolvida com a vida associativa na enfermagem por meio da Associação Brasileira de Enfermagem, seção Minas Gerais (ABEn-MG). Ocupou cargos no conselho fiscal, tais como de 23/06/1962 a 26/07/1964 e de 18/08/68 a 07/07/1970. Aposentou-se em 1970 pela EEUFMG, e faleceu em 08/05/2003.

Na Figura 1, são apontados os principais marcos da trajetória profissional de Rosa de Lima.

**Figura 1:** Linha do tempo profissional de Rosa de Lima Moreira.

Isaltina Goulart de Azevedo nasceu em 19 de março de 1917, em Itajubá, MG. Ainda em sua cidade natal, concluiu os cursos primário (1931) e normal (1936). Trabalhou por dez anos como professora, quando em 1947 ingressou na EECC.

Foi convidada a lecionar na instituição em 1949, antes mesmo de se formar, uma vez que não havia professores para ministrar a disciplina de "doenças transmissíveis". Para assumir tal responsabilidade, a diretora em exercício na EECC, Rosa de Lima Moreira, enviou Isaltina Goulart a Santos, São Paulo, para fazer um curso com prática clínica/estágio nesta temática.

Diplomada enfermeira em 1950, manteve a atuação como docente na EECC. Ingressou na Secretaria de Saúde e Assistência do estado de MG, com vinculação à Escola de Auxiliares de Enfermagem da Cruz Vermelha Brasileira-MG (1951 a 1962) e à ESP-MG, no curso de visitadora sanitária (1952 a 1956), ministrando temáticas nas áreas da história da enfermagem, ética, saúde pública e doenças transmissíveis.

Com o desejo de aprimorar as suas atividades como docente, cursou filosofia na UFMG, formando-se em 1955. Tinha uma vida profissional muito articulada: participou de congressos e ocupou cargos junto à ABEn. Em 1962, solicitou afastamento de suas atividades para realização de um curso de pós-graduação em enfermagem com especialidade em doenças transmissíveis, nos Estados Unidos, com bolsa financiada pela Fundação Rockefeller.

Ao retornar ao Brasil, em 1964, ampliou o escopo de articulações, ainda com o apoio da Fundação Rockefeller (instituição filantrópica que atuou desde o início do século XX no Brasil), com o Departamento de Medicina Preventiva da Faculdade de Medicina da UFMG e com a Organização Mundial da Saúde, lecionando e palestrando sobre o trabalho do enfermeiro. No mesmo ano, assumiu o cargo de chefe do Serviço de Enfermagem da Secretaria de Saúde de MG (1964 a 1968). Nesse período, organizou o Pavilhão de Doenças Infecciosas e Tropicais da Faculdade de Medicina da UFMG, e assumiu a coordenação de campanhas de vacinação no estado de MG, bem como inspecionou cursos de auxiliares de enfermagem no interior de MG, devido à preocupação em interiorizar as boas práticas de saúde.

Foi nomeada diretora da Escola de Enfermagem em novembro de 1968, para um mandato de três anos, sendo a primeira gestora da instituição a ter assento no Conselho Universitário. No período de 1950 a 1968, em que a EECC esteve anexada à Faculdade de Medicina da UFMG, a diretora do curso de enfermagem não possuía autonomia administrativa no âmbito da universidade, devendo direcionar todas as suas demandas ao diretor da medicina. Mesmo em um momento de subordinação da EECC, foi júri de concursos públicos e prêmios, e manteve atuação maciça em congressos de enfermagem.

Em julho de 1970, assumiu a presidência da ABEn-MG, permanecendo até 1972. Manteve sua articulação com aulas e palestras em saúde pública, ética e história da enfermagem entre a EE-UFMG/Faculdade de Medicina da UFMG/Hospital das Clínicas da UFMG/reitoria da UFMG/ESP-MG/Secretaria de Saúde de MG.

Em 1971, solicitou apoio para saída do país para estudos na Inglaterra, pois recebeu convite do Conselho Britânico, sendo apoiada com bolsa de estudos desta entidade. Também foi apoiada, em 1972, pela embaixada francesa, por solicitação da reitoria da UFMG, para conhecer serviços de enfermagem desses países. No ano seguinte, 1973, Isaltina Goulart participou do Congresso Nacional de Enfermagem em Portugal e proferiu a palestra de abertura "O papel do enfermeiro no mundo moderno", trazendo panoramas e perspectivas desse profissional nos países que havia visitado.

Ao retornar ao Brasil, Isaltina Goulart iniciou o investimento na pesquisa, com o projeto "Estudo da situação do Hospital das Clínicas da UFMG – como campo de treinamento para o pessoal da enfermagem". Tratava-se de proposta de peso para reorganização dos setores do hospital para atender às necessidades de campos de estágio para a EEUFMG. Ela partia do pressuposto de que a instituição, apesar de ser um Hospital Escola, não dispunha de condições mínimas estruturais para a aprendizagem da enfermagem. Assim, ela realizou um diagnóstico situacional dessas unidades, e as proposições de melhorias seriam baseadas em hospitais de ensino da Inglaterra, berço da institucionalização do ensino da enfermagem.

Em 1981, Isaltina Goulart foi designada, pelo Conselho Departamental da Escola de Enfermagem da UFMG, a exercer "*pro tempore*" na direção da referida unidade até a nomeação de um novo diretor.

Isaltina Goulart foi autora de textos científicos, podendo-se citar alguns exemplos, como o texto "A enfermagem e o enfermeiro", em que ela expõe falta de legitimação do campo da enfermagem e como isso contribui para que o preconceito em relação a essa profissão seja disseminado. Outro texto escrito por Isaltina em um evento em Portugal foi "O papel do enfermeiro no mundo moderno", no qual ela, mais uma vez, evoca a importância da enfermagem para a saúde brasileira. Tornou-se professora emérita em 1984. Faleceu em 15 de janeiro de 2009.

Os principais marcos da trajetória profissional de Isaltina Goulart estão representados na Figura 2.

**Figura 2:** Linha do tempo profissional de Isaltina Goulart.

Carmelita Pinto Rabelo nasceu em 16 de setembro de 1937, na Bahia, onde concluiu o curso normal, aos 19 anos. Em 1957, ingressou no curso de enfermagem da EECC. Ela concluiu o curso em janeiro de 1960 e, em fevereiro do mesmo ano, tornou-se docente na instituição.

A sua primeira atuação como docente foi na área da saúde pública, pois era a que mais necessitava de professores naquele momento da EECC. Ainda em 1960, fez estágio em Pirapora, MG, em unidade mista da função do Serviço Especial de Saúde Pública, instituição bilateral criada com os chamados Acordos de Washington (1942), com forte atuação na oferta de cursos e no auxílio aos serviços de saúde regionais, sobretudo nos estados de MG, Espírito Santo e Bahia. Essas vivências na saúde pública fizeram com que ela investisse em um curso de especialização em saúde pública na faculdade de higiene e saúde pública da Universidade de São Paulo (USP), em 1961. Em 1962, atuou como docente do curso de supervisoras sanitárias na ESP-MG.

Militante pela enfermagem, Carmelita Rabelo ocupou cargos na ABEn-MG, fez representações em órgãos colegiados da universidade, chefiou o Departamento de Enfermagem Materno-infantil e Saúde Pública da EEUFMG, participou de reformas curriculares, e envolveu-se com atividades estudantis e com projetos de extensão.

Em 1967, tornou-se a primeira diretora laica da EECC, após quase 20 anos de direções religiosas e subordinação administrativa à Faculdade de Medicina da UFMG. O exercício na diretoria lhe possibilitou articulações extramuros com a reitoria da UFMG para a desanexação da Escola de Enfermagem da Faculdade de Medicina e para o processo de autonomização da instituição após a Reforma Universitária brasileira de 1968.

Posteriormente, coordenou o primeiro vestibular para enfermagem da UFMG, pois o ingresso até então ocorria mediante processo simplificado e não unificado de provas. No período de 1968 a 1972, foi vice-diretora da Escola de Enfermagem, junto à Professora Isaltina Goulart de Azevedo, quando então a EECC foi denominada EEUFMG. Alguns anos depois, Carmelita concluiu, em 1985, o doutorado em saúde pública pela Faculdade de Saúde Pública da USP. Carmelita Pinto Rabelo se aposentou em 1991 e faleceu em 24 de setembro 2002.

Os principais achados profissionais da trajetória de Carmelita Rabelo estão representados na Figura 3.

**Figura 3:** Linha do tempo profissional de Carmelita Rabelo.

## DISCUSSÃO

As trajetórias de vida de Rosa de Lima Moreira, Isaltina Goulart de Azevedo e Carmelita Pinto Rabelo guardam singularidades nos universos feminino e profissional, assim como possuem diferenças de expressão nos construtos epistêmicos do ser/estar/fazer do enfermeiro.

No que se refere ao feminino, no contexto da Inglaterra vitoriana, a enfermagem se abre como uma oportunidade de carreira para a mulher branca, solteira e da alta sociedade. A institucionalização do conhecimento foi um diferencial para legitimar a profissionalização, conforme proposto por Florence Nightingale (1820–1910), mundialmente reconhecida como a precursora da enfermagem moderna^(16,17)^. O seu modelo de ensino e formação do enfermeiro foi reproduzido em todos os continentes.

Os impactos da institucionalização e internacionalização alcançados pelo modelo proposto por Florence Nightingale chegaram ao Brasil no início do século XX, com a Reforma Carlos Chagas que, em 1922, o incorporou à versão americana, que ficou conhecida como modelo anglo-americano. A materialização desse novo "estilo de pensamento" para a enfermagem ocorreu com a criação da Escola de Enfermeiras do Departamento Nacional de Saúde Pública, que deu origem, em 1923, à atual EEAN da UFRJ, e, subsequentemente, com a EECC, em 1933, atual EEUFMG^(18)^.

A tríade de mulheres legitimadoras da saúde pública e da enfermagem, aqui prosopografada, convergiu espacialmente na EECC em tempos históricos diferentes: Rosa de Lima está entre as estudantes pioneiras da EECC (década de 1930), além de Isaltina Goulart (década de 1940) e Carmelita Rabelo (década de 1950). Esses tempos refletem, respectivamente, alguns marcos: criação da escola sob a tutela do governo do estado de MG; equiparação ao padrão Anna Nery – modelo a ser seguido pelas escolas de enfermagem brasileiras criadas conforme as normativas do sistema anglo-americano (década de 1940); e federalização da instituição à UFMG, encampada e subordinada à Faculdade de Medicina. Cada uma dessas mulheres sedimenta uma etapa de contribuição científica e profissional para a consolidação da enfermagem, indicando um processo não linear e com tensões singulares aos tempos e contextos vivenciados por elas e sua categoria.

A despeito dos tempos históricos diversos, a socialização acadêmica e profissional dessas enfermeiras aconteceu na mesma instituição, com valores semelhantes e a busca de um *ethos* para a enfermagem. Certamente, isso contribuiu para forjar coesão intelectual e ideológica para frentes de trabalho comuns, como construção e expansão de sua área de conhecimento (a enfermagem) somada à sua responsabilidade social com a saúde pública.

Do ponto de vista epistemológico, a enfermagem se constitui em uma prática que se transforma a partir das mudanças da sociedade^(19)^. Como apontado, esse campo tensiona o processo de profissionalização, conformando novas práticas que exigirão adaptações à formação e constantes capacitações para que se tenha uma enfermeira que atenda requisitos formais e legais, atentas às necessidades colocadas historicamente, do seu grupo e das suas coletividades. Como produz conhecimentos, trazendo respostas às diferentes necessidades de saúde da população, a enfermagem é uma ciência em que a práxis e a crítica devem fazer parte do cotidiano profissional.

Seja pela responsabilidade social, seja pela/para a enfermagem mineira, observa-se um movimento temporal e geracional em que uma enfermeira prepara o campo para a outra: um passo dado para que outras continuem. Desse modo, são produzidos movimentos articulados e coletivos em torno da enfermagem.

Rosa de Lima era farmacêutica e proprietária de uma farmácia, tornando-se estudante de enfermagem e professora ainda na década de 1930. Ela assumiu cargos de alta liderança na instituição e em momentos de alta vulnerabilidade, como quando ocorreu a saída da diretora da EECC (1940–1948): Waleska Paixão. Rosa de Lima era uma personagem com uma visão acurada das demandas à época e de como a enfermagem poderia contribuir com um movimento recíproco de fortalecimento da saúde pública e na constituição do seu próprio campo profissional. Sua continuidade nesses espaços de gestão permite que a EECC exista/resista para que Isaltina Goulart, a segunda mulher da tríade, possa ser estudante e abrir frente para um trabalho conjunto à Carmelita Rabelo de laicização (a EECC foi dirigida por irmãs de caridade no período 1950 a 1968). O fim da década de 1960 e início dos anos 1970 mostram a dupla Isaltina/Carmelita protagonizando a primeira gestão laica desta instituição após a Reforma Universitária de 1968, que incorporou, entre outras frentes, a pesquisa como parte da vida universitária. A partir deste fato, ambas permitem que a EECC, redenominada EEUFMG, passasse a ter assento no Conselho Universitário.

Mas as atuações e trajetórias não se reduzem apenas à EECC/EEUFMG. Como mulheres atentas ao tempo histórico, as três enfermeiras ocuparam posições de poder em diferentes estruturas políticas e burocráticas, como ESP-MG (fundada em 1946) e Secretaria do Estado de MG, financiadas por agências para cursos de profissionalização em outros estados brasileiros e fora do país. Essas interlocuções denotam o alcance da *expertise* dessas agentes sociais junto à saúde pública mineira, demonstrando como ampliaram o *status quo* das enfermeiras em seus feitos.

Outro ponto a ser observado é a aderência da tríade quanto ao associativismo na enfermagem. A prerrogativa de uma profissão forte após a implantação do modelo anglo-americano no Brasil previa a existência de uma associação para que as enfermeiras pudessem congregar seus interesses. Assim, em 1936, foi criada a Associação Nacional de Enfermeiras Diplomadas, que deu origem à atual ABEn^(20)^.

Junto a isso, nota-se uma preocupação em atualizar a enfermagem, para além das práticas instituídas, como um campo de pesquisas. É algo que se vê na disposição e dedicação de Isaltina Goulart em dar os primeiros passos em direção à produção de conhecimento e pesquisa em enfermagem. Isaltina liderou os primeiros projetos de pesquisa que pensavam a enfermagem como o caminho para a melhoria da saúde pública no estado^(21,22)^.

Ao evidenciar a trajetória da tríade de mulheres que legitima a saúde pública mineira e a enfermagem, são vistos empreendimentos robustos para alçar a enfermagem a outros patamares, maiores e mais afinados com as expectativas do ambiente universitário a partir da década de 1960. Dessa forma, tem-se a nitidez que cada uma, em seu tempo histórico, contribuiu com diferentes formas para um processo maior que envolve a enfermagem brasileira. Ao notabilizar o percurso vivenciado por elas, três espaços emblemáticos da saúde e da profissionalização da enfermagem aparecem de forma recorrente (Figura 4): EECC, ESP-MG, ABEn-MG.

**Figura 4:** Consonância entre as atrizes sociais da medicina tropical.

A combinação da prosopografia com outros métodos pode ser empreendida para ampliar o entendimento das dinâmicas sociais. Dessa forma, o presente estudo apresenta informações inéditas sobre três mulheres, enfermeiras e vanguardistas no campo da enfermagem em saúde pública. O cruzamento das trajetórias mostra que a tríade de enfermeiras mineiras buscou legitimidade para a enfermagem no âmbito acadêmico e na vida associativa em prol da saúde pública, contribuindo para uma inflexão profissional e epistemológica do coletivo de pensamento neste campo e na saúde.

As limitações do estudo envolveram a falta de informações sistemáticas sobre determinados momentos das carreiras das atrizes sociais prosopografadas, principalmente no que se refere às suas participações na ESP-MG. Acredita-se que a não preservação de fontes dessa coleção documental ocasionou a perda de informações valiosas sobre suas atuações fora do circuito EECC/EEUFMG e ABEn. Contudo, assumidas as possíveis generalizações, realça-se o esforço dessas enfermeiras em crescer e valorizar a enfermagem na saúde pública.

## CONCLUSÃO

A trajetória de Rosa de Lima Moreira, Isaltina Goulart de Azevedo e Carmelita Pinto Rabelo nos ajuda a compreender que os processos de inserção de mulheres no campo da saúde pública no Brasil se iniciam de forma mais sistemática no pós-segunda guerra mundial, no final dos anos 1940. Naquela época, as mulheres ingressavam ainda muito jovens e antes mesmo de terminarem os estudos regulares. Outro elemento importante no processo de profissionalização da tríade no campo da medicina tropical são as instituições públicas e os serviços como a ESP-MG e institutos. Na passagem pelos diferentes institutos, as mulheres "fizeram de tudo", ocupando as mais variadas atividades técnicas e de produção intelectual.

# Translation: Análisis fleckiano del enfermero de salud pública en la legitimación de la profesión en Minas Gerais (1940–1970)

**DOI:** 10.1590/1980-220X-REEUSP-2024-0427es

**Authors:** Bráulio Silva Chaves, Isabelle de Souza Januaria, Fernanda Batista Oliveira Santos

**Affiliations:** 1 Centro Federal de Educação Tecnológica de Minas, Departamento de Ciências Sociais e Filosofia, Belo Horizonte, MG, Brasil.; 2 Fiocruz Minas, Instituto René Rachou, Belo Horizonte, MG, Brasil.; 3 Universidade Federal de Minas Gerais, Escola de Enfermagem, Programa de Pós-Graduação em Enfermagem, Belo Horizonte, MG, Brasil.

**Keywords:** Rasgos de la Historia de Vida, Historia de la Enfermería, Enfermería en Salud Pública, Procesos de Grupo, Empleos en Salud

## Abstract

**Objetivo:**

Analizar la trayectoria de tres enfermeras – Rosa de Lima Moreira, Isaltina Goulart de Azevedo y Carmelita Pinto Rabelo –, considerando sus acciones en Minas Gerais para legitimar el pensamiento colectivo de enfermería, a partir de las ideas de Fleck, entre 1940 y 1970.

**Método:**

Estudio en el campo de la historia de la ciencia en interfaz con la historia de la enfermería cualitativa y documental. Se recopilaron fuentes de diferente tipología y naturaleza prosopográfica. El análisis consideró la referencia de Ludwik Fleck.

**Resultados:**

Los hallazgos históricos indican un fuerte movimiento de estas mujeres en espacios institucionales educativos y asociativos a favor de la salud pública en Minas Gerais, con agendas para la formación de cuadros técnicos profesionales en educación para la salud en la Escuela de Salud Pública de Minas Gerais, en espacios dentro del alcance de los cursos de graduación y posgrado de la Escuela de Enfermería de la Universidade Federal de Minas Gerais y el activismo por la profesión en la Asociación Brasileña de Enfermería.

**Conclusión:**

El cruce de trayectorias muestra que la tríada de enfermeros de Minas Gerais buscó legitimar la enfermería, contribuyendo a una inflexión profesional y epistemológica.

## INTRODUCCIÓN

La grave situación de la salud pública en Brasil en la década de 1920 llevó a Carlos Chagas, director del Departamento Nacional de Salud Pública, a buscar en Estados Unidos un modelo de formación de enfermería que pudiera hacer frente a la situación, basado en la educación sanitaria. En 1923, se creó en la entonces capital federal, Río de Janeiro, la Escuela del Departamento Nacional de Salud Pública, que dio origen a la actual Escuela de Enfermería Anna Nery (EEAN) de la Universidad Federal de Río de Janeiro (UFRJ)^(1)^. En la estela de este movimiento de profesionalización en suelo nacional, se creó en 1933 la Escuela de Enfermería Carlos Chagas (EECC), actual Escuela de Enfermería de la Universidad Federal de Minas Gerais (EEUFMG)^(2)^, donde estudiaron y trabajaron Rosa de Lima Moreira, Isaltina Goulart de Azevedo y Carmelita Pinto Rabelo, la tríada de enfermeras de esta investigación.

El contexto social, político y económico brasileño del siglo XX estuvo marcado por la inestabilidad democrática y por momentos de fuerte autoritarismo estatal. Estos aspectos influyeron en la forma en que se organizó el sector sanitario, especialmente desde el punto de vista de la salud^(3)^. Así, ganaron protagonismo profesionales centrados en la educación de la población, como la enfermera de salud pública y otras estratificaciones técnicas, como los visitadores y educadores sanitarios y los coordinadores de salud escolar^(4)^. Cabe destacar que, hasta la década de 1940, la tarea de formar profesionales de educación para la salud en Brasil era ejercida principalmente por médicos. Sin embargo, este escenario cambió con la influencia de una agenda internacional propagada por la Organización Mundial de la Salud^(5)^.

La década de 1960 estuvo particularmente marcada por los militares (1964) en el centro de las decisiones políticas. En lo que respecta al sistema sanitario, el país experimentó una duplicidad entre la asistencia de la seguridad social y la sanidad pública. La primera se centraba en la salud individual de los trabajadores formales, mientras que la segunda, bajo el mando del Ministerio de Sanidad, se dirigía principalmente a las zonas rurales y a los sectores más pobres de la población, y se centraba sobre todo en actividades preventivas^(6)^.

Aunque la EEAN y la EECC se crearon en este contexto de diferentes demandas de salud pública en Brasil, los intereses de las enfermeras que dirigían estas instituciones se centraban no sólo en este campo, sino también en otros^(7)^. Es decir, les interesaba legitimar la profesión de enfermería en sus diversas posibilidades y diferentes ámbitos de actuación, como escuelas, centros de salud, hospitales, empresas, *etc.* ^(2)^.

En concreto, la EECC era una institución financiada por el gobierno del estado de Minas Gerais (MG)^(8)^. Esta situación fue diferente a la de la EEAN, que pronto se federalizó y pasó a formar parte de lo que luego sería la UFRJ^(7)^. A lo largo de sus primeros 17 años de funcionamiento, la EECC tuvo muchas dificultades para graduar un número suficiente de estudiantes para satisfacer las demandas de salud pública del estado de Minas Gerais. Esas dificultades se explican por diversos factores, entre ellos: la situación de la mujer en la sociedad brasileña de la época, que aún tenía pocas oportunidades de inserción en el mercado de trabajo; la duración del curso, de tres años; y el escaso atractivo de la carrera de enfermera en ese modelo de formación^(8,9)^. Así, la EECC enfrentó una grave crisis al final de la década de 1940, careciendo de las condiciones mínimas para su mantenimiento. El gobierno de MG abandonó progresivamente la EECC, culminando con la creación de la Escuela de Salud Pública de Minas Gerais (ESP-MG), que ofrecía formación a través de cursos cortos (de 3 a 6 meses), como el de educadores sanitarios, que satisfacía pragmáticamente algunas demandas inmediatas de personal técnico^(9)^.

En este contexto, destacan tres nombres en la enfermería de Minas Gerais surgidos entre los años 40 y 70: Rosa de Lima Moreira, Isaltina Goulart de Azevedo y Carmelita Pinto Rabelo. Las tres se licenciaron en enfermería en la EEUFMG, actuaron en la sanidad pública y ocuparon cargos en la dirección de la institución, y son reconocidas por su importante papel en la formación y profesionalización de la enfermería para el estado y Brasil^(10)^. Las tres tienen también un punto de convergencia en sus carreras, que fue su trabajo en cursos de formación en educación para la salud en la ESP-MG^(11)^. Esos puntos de convergencia plantearon las siguientes cuestiones: ¿quiénes son esas mujeres que empezaron a asumir funciones de profesoras y coordinadoras de cursos de educación para la salud, promoviendo la profesionalización de la enfermería? ¿De dónde venían, cuál era su formación y su perfil laboral? ¿Hasta qué punto es posible, a partir de sus trayectorias, establecer una red de circulación dentro de las estructuras de salud pública, así como en otros espacios académicos, como la EEUFMG y la ESP-MG? ¿Cómo sus vidas profesionales ayudan a construir parte de la historia de la enfermería en el período analizado?

Se asume que una mirada a la trayectoria de estas mujeres contribuyó a una mayor dimensión en la historia de la enfermería y sus recorridos, a través de otras variables, como la local, regional, contemplando las aproximaciones con otros colectivos como escenarios definitorios de cambio para la salud y para la propia enfermería.

En este sentido, Ludwik Fleck, epistemólogo polaco, reflexiona sobre cómo se forma un «colectivo de pensamiento» a partir de un conjunto de acciones de sujetos en diversas esferas sociales, contribuyendo a pensar este proceso para la enfermería. Fleck ayuda a problematizar los movimientos dentro y fuera de la enfermería asociados a los cambios en los modelos de salud que marcaron la época posterior a la Segunda Guerra Mundial, reconfigurando la enfermería como profesión^(12)^.

Se trata de nuevos «estilos de pensar» que actualizan las prácticas de enfermería ante el escenario inestable de Brasil y las demandas internacionales de salud, en diálogo con aspectos regionales que marcan la especificidad de las instituciones. Para estos análisis, consideramos las lagunas existentes en lo que se refiere a una mirada más completa sobre la importancia de las mujeres en la historia de la ciencia y de la salud pública en el estado de Minas Gerais.

El objetivo de este estudio es, por lo tanto, analizar la trayectoria de tres enfermeras – Rosa de Lima Moreira, Isaltina Goulart de Azevedo y Carmelita Pinto Rabelo – considerando su trabajo en Minas Gerais para legitimar el pensamiento colectivo de la enfermería, basado en las ideas de Fleck, entre 1940 y 1970.

## MÉTODO

### Diseño Del Estudio

Se trata de un estudio en el campo de la historia de las ciencias en interfaz con la historia de la enfermería^(13)^. Como abordaje, se utilizó el *COnsolidated criteria for REporting Qualitative research* (COREQ), versión en portugués brasileño^(14)^, basado en fuentes documentales y prosopografía, partiendo de biografías individuales para componer una biografía social^(15)^. El análisis se basó en el pensamiento epistemológico de Ludwik Fleck^(12)^.

### Lugar

El escenario del estudio comprende los lugares en los que trabajó la tríada de profesores de salud pública en los decenios de 1940 y 1970. Este marco temporal incluye el período en que se ofrecieron cursos sobre educación sanitaria en la ESP-MG (el primero en 1947 y el último en 1968 – el período de la medicina tropical), y cuando los profesores trabajaron en la EEUFMG. Los dos espacios principales son las instituciones de enseñanza en salud de Belo Horizonte, MG, como la ESP-MG y la EECC, que dio origen a la EEUFMG.

### Criterios de Selección

La ESP-MG y el Centro de Memoria de la Escuela de Enfermería de la UFMG (CEMENF) poseen un acervo documental sobre cursos de formación, que fue seleccionado para esta investigación.

La documentación fue abordada de forma cualitativa, teniendo en cuenta algunas de las inferencias de la investigación: la importancia de los cursos de educación para la salud como espacios de ampliación del papel de la mujer y destacando a las enfermeras que trabajan en salud pública; los movimientos emprendidos por las mujeres en las instituciones de salud de Minas Gerais y cómo impactaron en la consolidación de la enfermería en el período estudiado; la trayectoria de tres mujeres que ejemplifican el papel de la enfermería en salud pública en la formación en diversos niveles; y el abordaje de la investigación y del posgrado en las asociaciones y entidades gestoras.

El acceso a las fuentes siguió los siguientes pasos 1) recopilación de fuentes en ESP-MG sobre los cursos de educación sanitaria (visitadoras sanitarias y coordinadora de salud escolar) entre los años 40 y 60; 2) organización de la documentación y su estratificación, utilizando la tabulación de datos para componer el escenario; 3) recorte de las mujeres que trabajaron como profesoras y coordinadoras de los cursos; 4) cruce con la base de datos del CEMENF, colección Carlos Chagas, para saber qué profesoras y coordinadoras eran diplomadas en enfermería por la EEUFMG; 5) recogida de datos sobre el movimiento de estas mujeres en las instituciones y en la gestión sanitaria, a partir de la documentación encontrada; 6) selección de tres mujeres para la investigación, por su importancia en el colectivo de enfermería y en la salud pública, teniendo en cuenta que ocuparon cargos docentes, de gestión e incluso de dirección en la EEUFMG.

### Recogida de Datos

Los datos se recogieron en la EEUFMG de marzo a agosto de 2021, y en el CEMENF de octubre de 2023 a marzo de 2024.

### Análisis y Tratamiento de los Datos

Los hallazgos de las trayectorias de las actrices sociales prosopografiadas se analizaron dentro del marco propuesto por Fleck, en el que las biógrafas son sujetos sociales que reflejan un «colectivo de pensamiento» en enfermería en el periodo estudiado y la correlación entre ellos y los nuevos «estilos de pensamiento» en las ciencias de la salud^(12)^.

### Aspectos Éticos

El estudio se realizó de acuerdo con las directrices éticas nacionales e internacionales. El *corpus* documental se constituyó mediante búsquedas en dos colecciones públicas, sin conflicto de intereses y sin necesidad de dictamen del Comité de Ética en Investigación, según lo dispuesto en las Resoluciones 466/2012 y 510/2016 del Consejo Nacional de Salud.

## RESULTADOS

### Tres Mujeres que Legitimaron la Enfermería en Diferentes Frentes y de Forma Cruzada

Rosa de Lima Moreira nació el 30 de agosto de 1905 en el interior de Minas Gerais. Vivió la mayor parte de su vida en la ciudad de Belo Horizonte, capital del estado, con su familia, propietaria de una farmacia en el barrio de Horto. Bajo la influencia de su padre, a los 16 años ingresó en el curso de farmacia de la Escuela de Odontología y Farmacia de Belo Horizonte. En 1921, se graduó como farmacéutica y heredó la farmacia de su padre. Aún bajo la influencia paterna, ingresó en la segunda promoción de enfermería de la EECC.

Se graduó en 1936 y fue invitada a convertirse en instructora de la institución. Entre 1939 y 1948, participó en la dirección de la escuela como subdirectora y, entre 1948 y 1950, como directora de la EECC. En 1950, ya no como directora, Rosa de Lima fue testigo del proceso de federalización de la EECC.

Posteriormente, fue profesora de la ESP-MG en el curso de visitadora médica (1953–1955), impartiendo asignaturas en las áreas de historia y ética de la enfermería. En marzo de 1954, Rosa fue invitada de nuevo a hacerse cargo de la junta directiva durante la administración de las Hermanas de la Caridad. Permaneció en el cargo hasta marzo de 1955.

En 1960 y 1961, realizó una pasantía en Bahia, subvencionada por una beca de pasantía en medicina clínica en la Escuela de Enfermería de la Universidad Federal de Bahia. En octubre de 1961, visitó la EEAN para actualizar sus estudios sobre el escenario de la clínica médica en la ciudad de Río de Janeiro. Durante esta visita, Rosa de Lima fue invitada por la directora de la EEAN, Waleska Paixão, a participar en un curso de extensión sobre el mismo tema.

En 1968, con la Reforma Universitaria, liderada dentro de la EECC por las profesoras Isaltina Goulart y Carmelita Rabelo, fue testigo del proceso de secularización y de la reconfiguración de la EECC en la EEUFMG.

Rosa de Lima siempre estuvo implicada en las asociaciones de enfermería a través de la Asociación Brasileña de Enfermería, sección Minas Gerais (ABEn-MG). Ocupó cargos en el consejo de vigilancia del 23/06/1962 al 26/07/1964 y del 18/08/68 al 07/07/1970. Se jubiló en 1970 de la EEUFMG y falleció el 08/05/2003.

La Figura 1 muestra los principales hitos de la carrera profesional de Rosa de Lima.

**Figura 1:** Trayectoria profesional de Rosa de Lima Moreira.

Isaltina Goulart de Azevedo nació el 19 de marzo de 1917 en Itajubá, MG. En su ciudad natal, terminó la enseñanza primaria (1931) y la normal (1936). Trabajó como profesora durante diez años cuando ingresó en la EECC en 1947.

Fue invitada a enseñar en la institución en 1949, incluso antes de graduarse, ya que no había profesores para impartir la asignatura de «enfermedades transmisibles». Para asumir esta responsabilidad, la directora en funciones del EECC, Rosa de Lima Moreira, envió a Isaltina Goulart a Santos, São Paulo, para realizar un curso con prácticas clínicas en esta materia.

Se graduó como enfermera en 1950 y continuó enseñando en la EECC. Se incorporó al Departamento de Salud y Asistencia del estado de Minas Gerais, trabajando en la Escuela de Auxiliares de Enfermería de la Cruz Roja Brasileña (1951 a 1962) y en la ESP-MG, como visitadora sanitaria (1952 a 1956), impartiendo asignaturas en las áreas de historia de la enfermería, ética, salud pública y enfermedades transmisibles.

Con el deseo de mejorar sus aptitudes pedagógicas, estudió filosofía en la UFMG, donde se licenció en 1955. Tuvo una vida profesional muy articulada: participó en congresos y ocupó cargos en la ABEn. En 1962, pidió una excedencia para asistir a un curso de posgrado en enfermería, especializado en enfermedades transmisibles, en Estados Unidos, con una beca financiada por la Fundación Rockefeller.

A su regreso a Brasil, en 1964, amplió el alcance de su trabajo, siempre con el apoyo de la Fundación Rockefeller (institución filantrópica que actuaba en Brasil desde principios del siglo XX), con el Departamento de Medicina Preventiva de la Facultad de Medicina de la UFMG y con la Organización Mundial de la Salud, impartiendo clases y conferencias sobre el trabajo de las enfermeras. En el mismo año, pasó a dirigir el Servicio de Enfermería del Departamento de Salud de la MG (1964 a 1968). Durante este período, organizó el Pabellón de Enfermedades Infecciosas y Tropicales de la Facultad de Medicina de la UFMG, y asumió la coordinación de las campañas de vacunación en el estado de MG, así como la inspección de los cursos para auxiliares de enfermería en el interior de MG, debido a su preocupación por interiorizar las buenas prácticas sanitarias.

Fue nombrada directora de la Escuela de Enfermería en noviembre de 1968, por un mandato de tres años, y fue la primera mujer en formar parte del Consejo Universitario. De 1950 a 1968, cuando el EECC estaba adscrito a la Facultad de Medicina de la UFMG, la directora de la carrera de enfermería no tenía autonomía administrativa dentro de la universidad, y debía dirigir todas sus demandas al director de medicina. Incluso en la época en que la EECC estaba subordinada, fue jurado de concursos públicos y premios, y mantuvo una presencia masiva en los congresos de enfermería.

En julio de 1970 se convirtió en presidente de la ABEn-MG, cargo que ocupó hasta 1972. Continuó organizando clases y conferencias sobre salud pública, ética e historia de la enfermería entre EE-UFMG/Escuela de Medicina de UFMG/Hospital das Clínicas de UFMG/Rectorado de UFMG/ESP-MG/Departamento de Salud de UFMG.

En 1971, solicitó apoyo para salir del país para estudiar en Inglaterra, ya que recibió una invitación del British Council y fue apoyada con una beca de esta organización. En 1972, también fue apoyada por la embajada de Francia, a petición de la rectoría de la UFMG, para conocer los servicios de enfermería en esos países. Al año siguiente, 1973, Isaltina Goulart participó en el Congreso Nacional de Enfermería en Portugal y dio la conferencia de apertura «El papel de la enfermera en el mundo moderno», presentando panoramas y perspectivas de esta profesional en los países que había visitado.

A su regreso a Brasil, Isaltina Goulart comenzó a invertir en investigación, con el proyecto «Estudio de la situación del Hospital das Clínicas de la UFMG – como espacio de formación para el personal de enfermería». Se trataba de una importante propuesta de reorganización de los sectores del hospital para responder a las necesidades de la EEUFMG como lugar de formación. Partía de la base de que la institución, a pesar de ser un hospital escuela, no reunía las condiciones estructurales mínimas para el aprendizaje de la enfermería. Por ello, realizó un diagnóstico de situación de estas unidades y propuso mejoras basadas en los hospitales docentes de Inglaterra, cuna de la institucionalización de la enseñanza de la enfermería.

En 1981, Isaltina Goulart fue nombrada por el Consejo Departamental de la Escuela de Enfermería de la UFMG para dirigir la unidad «*pro tempore»* hasta el nombramiento de un nuevo director.

Isaltina Goulart fue autora de varios textos científicos, entre ellos el texto «A enfermagem e o enfermeiro» (La enfermería y el enfermero), en el que expone la falta de legitimación del campo de la enfermería y cómo esto contribuye a la propagación de prejuicios contra esta profesión. Otro texto escrito por Isaltina en un acto en Portugal fue «El papel de la enfermera en el mundo moderno», en el que vuelve a evocar la importancia de la enfermería para la salud brasileña. Se convirtió en profesora emérita en 1984. Falleció el 15 de enero de 2009.

En la Figura 2 se muestran los principales hitos de la carrera profesional de Isaltina Goulart.

**Figura 2:** Cronología profesional de Carmelita Rabelo.

Carmelita Pinto Rabelo nació el 16 de septiembre de 1937 en Bahía, donde completó el curso normal a los 19 años. En 1957, ingresó en el curso de enfermería de la EECC. Terminó el curso en enero de 1960 y, en febrero del mismo año, se convirtió en docente de la institución.

Su primera actuación como docente fue en el área de salud pública, ya que era la que más necesitaba profesores en ese momento en la EECC. Aún en 1960, realizó una pasantía en Pirapora, MG, en una unidad mixta del Servicio Especial de Salud Pública, institución bilateral creada en los llamados Acuerdos de Washington (1942), con una fuerte actuación en la oferta de cursos y en el apoyo a los servicios de salud regionales, sobre todo en los estados de MG, Espírito Santo y Bahía. Estas experiencias en salud pública la llevaron a invertir en un curso de especialización en salud pública en la Facultad de Higiene y Salud Pública de la Universidad de São Paulo (USP) en 1961. En 1962, actuó como docente en el curso de supervisoras sanitarias en la ESP-MG.

Militante por la enfermería, Carmelita Rabelo ocupó cargos en la ABEn-MG, representó en órganos colegiados de la universidad, dirigió el Departamento de Enfermería Materno-Infantil y Salud Pública de la EEUFMG, participó en reformas curriculares, se involucró en actividades estudiantiles y en proyectos de extensión.

En 1967, se convirtió en la primera directora laica de la EECC, después de casi 20 años de direcciones religiosas y subordinación administrativa a la Facultad de Medicina de la UFMG. Su ejercicio en la dirección le permitió articularse extramuros con el rectorado de la UFMG para la desvinculación de la Escuela de Enfermería de la Facultad de Medicina y el proceso de autonomía de la institución tras la Reforma Universitaria brasileña de 1968.

Posteriormente, coordinó el primer examen de ingreso para enfermería en la UFMG, ya que hasta entonces el ingreso se realizaba mediante un proceso simplificado y no unificado de pruebas. Entre 1968 y 1972, fue vicedirectora de la Escuela de Enfermería, junto con la profesora Isaltina Goulart de Azevedo, cuando la EECC pasó a denominarse EEUFMG. Algunos años después, en 1985, Carmelita concluyó el doctorado en salud pública en la Facultad de Salud Pública de la USP. Se jubiló en 1991 y falleció el 24 de septiembre de 2002.

Los principales hitos profesionales de la trayectoria de Carmelita Rabelo están representados en la Figura 3.

**Figura 3:** Línea de tiempo profesional de Carmelita Rabelo.

## DISCUSIÓN

Las trayectorias vitales de Rosa de Lima Moreira, Isaltina Goulart de Azevedo y Carmelita Pinto Rabelo presentan singularidades en los universos femenino y profesional, así como diferencias de expresión en las construcciones epistémicas del ser/ser/hacer de las enfermeras.

En lo que respecta a las mujeres, en el contexto de la Inglaterra victoriana, la enfermería se abrió como una oportunidad profesional para las mujeres blancas, solteras y de la alta sociedad. La institucionalización del conocimiento fue un factor clave para legitimar la profesionalización, tal y como propuso Florence Nightingale (1820–1910), reconocida mundialmente como la precursora de la enfermería moderna^(16,17)^. Su modelo de enseñanza y formación de enfermeras se reprodujo en todos los continentes.

Los impactos de la institucionalización e internacionalización alcanzados por el modelo propuesto por Florence Nightingale llegaron a Brasil a principios del siglo XX, con la Reforma Carlos Chagas, que en 1922 lo incorporó a la versión americana, que pasó a ser conocida como modelo angloamericano. La materialización de este nuevo «estilo de pensar» para la enfermería ocurrió con la creación de la Escuela de Enfermeras del Departamento Nacional de Salud Pública, que dio origen en 1923 a la actual EEAN en la UFRJ, y posteriormente con la EECC en 1933, a la actual EEUFMG^(18)^.

La tríada de mujeres que legitimaron la salud pública y la enfermería, aquí descrita, convergió espacialmente en la EECC en diferentes momentos de la historia: Rosa de Lima figura entre las alumnas pioneras de la EECC (década de 1930), así como Isaltina Goulart (década de 1940) y Carmelita Rabelo (década de 1950). Estas épocas reflejan, respectivamente, algunos hitos: la creación de la escuela bajo la tutela del gobierno del estado de Minas Gerais; la equivalencia al estándar Anna Nery - modelo a seguir por las escuelas de enfermería brasileñas creadas según las normas del sistema angloamericano (década de 1940); y la federalización de la institución a la UFMG, asumida y subordinada a la Facultad de Medicina. Cada una de ellas estableció una etapa de contribución científica y profesional para la consolidación de la enfermería, indicando un proceso no lineal con tensiones propias de los tiempos y contextos vividos por ellas y su categoría.

A pesar de los diferentes momentos históricos, la socialización académica y profesional de estas enfermeras tuvo lugar en la misma institución, con valores similares y la búsqueda de un *ethos* para la enfermería. Sin duda, esto contribuyó a forjar una cohesión intelectual e ideológica en frentes comunes de trabajo, como la construcción y ampliación de su área de conocimiento (enfermería), además de su responsabilidad social con la salud pública.

Desde el punto de vista epistemológico, la enfermería es una práctica que se transforma con los cambios de la sociedad^(19)^. Como se ha señalado, este campo presiona sobre el proceso de profesionalización, configurando nuevas prácticas que requerirán adaptaciones en la formación y cualificación constante para que tengamos una enfermera que cumpla con los requisitos formales y legales, atenta a las necesidades históricamente situadas de su grupo y colectividades. Al producir conocimiento, dando respuesta a las diferentes necesidades de salud de la población, la enfermería es una ciencia en la que la praxis y la crítica deben formar parte del día a día profesional.

Ya sea por responsabilidad social o por/para la enfermería en Minas Gerais, existe un movimiento temporal y generacional en el que una enfermera prepara el campo para otra: un paso dado para que otras continúen. De este modo, se producen movimientos articulados y colectivos en torno a la enfermería.

Rosa de Lima era farmacéutica y propietaria de una farmacia que se convirtió en estudiante y profesora de enfermería en la década de 1930. Ocupó cargos de responsabilidad en la institución y en momentos de gran vulnerabilidad, como cuando se fue el director de la EECC (1940–1948): Waleska Paixão. Rosa de Lima fue un personaje con una visión certera de las demandas de la época y de cómo la enfermería podía contribuir a un movimiento recíproco de fortalecimiento de la salud pública y de constitución de un campo profesional propio. Su continuidad en estos espacios de gestión permitió la existencia de la EECC para que Isaltina Goulart, la segunda mujer de la tríada, pudiera ser alumna y abrir camino al trabajo conjunto con Carmelita Rabelo sobre la secularización (la EECC fue dirigida por hermanas de la caridad de 1950 a 1968). A finales de los sesenta y principios de los setenta, el dúo Isaltina/Carmelita lideró la primera gestión laica de esta institución tras la Reforma Universitaria de 1968, que incorporó la investigación como parte de la vida universitaria, entre otras cosas. Gracias a ello, ambas permitieron que la EECC, rebautizada como EEUFMG, tuviera un puesto en el Consejo de la Universidad.

Pero su trabajo y su trayectoria no se limitaron a la EECC/EEUFMG. Como mujeres atentas a los tiempos históricos, las tres enfermeras ocuparon puestos de poder en diferentes estructuras políticas y burocráticas, como la ESP-MG (fundada en 1946) y la Secretaría de Estado de MG, financiadas por agencias para cursos de profesionalización en otros estados brasileños y en el extranjero. Estas interlocuciones muestran el alcance de la *pericia de* estos agentes sociales en la salud pública de Minas Gerais, demostrando cómo ampliaron el *status quo* de las enfermeras en su empeño.

Otro punto a destacar es la adhesión de la tríada a las asociaciones de enfermería. La prerrogativa de una profesión fuerte tras la implantación del modelo angloamericano en Brasil exigía la existencia de una asociación para que las enfermeras pudieran poner en común sus intereses. Así, en 1936, se creó la Asociación Nacional de Enfermeras Graduadas, que dio origen a la actual ABEn^(20)^.

Paralelamente, existía la preocupación de actualizar la enfermería más allá de las prácticas establecidas como campo de investigación. Esto se puede ver en la voluntad y dedicación de Isaltina Goulart para dar los primeros pasos hacia la producción de conocimiento e investigación en enfermería. Isaltina lideró los primeros proyectos de investigación que veían en la enfermería la forma de mejorar la salud pública en el estado^(21,22)^.

Al resaltar la trayectoria de la tríada de mujeres que legitimaron la salud pública en Minas Gerais y la enfermería, podemos ver emprendimientos robustos para elevar la enfermería a nuevas alturas, más altas y en sintonía con las expectativas del ambiente universitario a partir de la década de 1960. De esta forma, queda claro que cada una, en su momento histórico, contribuyó de forma diferente a un proceso mayor que involucró a la enfermería brasileña. Al destacar el recorrido vivido, aparecen de forma recurrente tres espacios emblemáticos de la salud y de la profesionalización de la enfermería (Figura 4): EECC, ESP-MG, ABEn-MG.

**Figura 4:** Consonancia entre las actrices sociales de la medicina tropical.

La combinación de la prosopografía con otros métodos puede servir para ampliar nuestra comprensión de las dinámicas sociales. De este modo, este estudio presenta información inédita sobre tres mujeres, enfermeras y vanguardistas en el campo de la enfermería de salud pública. La intersección de sus trayectorias muestra que la tríada de enfermeras de Minas Gerais buscó la legitimación de la enfermería en el ámbito académico y en la vida asociativa a favor de la salud pública, contribuyendo a una inflexión profesional y epistemológica del colectivo de pensamiento en este campo y en la salud.

Las limitaciones del estudio se refieren a la falta de información sistemática sobre determinados momentos de la trayectoria de los actores sociales prosopografiados, especialmente en lo que se refiere a su participación en la ESP-MG. Se cree que la falta de preservación de las fuentes de ese acervo documental hizo que se perdiera información valiosa sobre su trabajo fuera del circuito EECC/EEUFMG y ABEn. Sin embargo, suponiendo que las generalizaciones sean posibles, vale la pena destacar los esfuerzos de estas enfermeras para hacer crecer y mejorar la enfermería de salud pública.

## CONCLUSIÓN

Las trayectorias de Rosa de Lima Moreira, Isaltina Goulart de Azevedo y Carmelita Pinto Rabelo nos ayudan a comprender que el proceso de entrada de las mujeres en el campo de la salud pública en Brasil comenzó de forma más sistemática después de la Segunda Guerra Mundial, a finales de la década de 1940. En aquella época, las mujeres se incorporaron a la profesión a una edad muy temprana e incluso antes de haber finalizado sus estudios regulares. Otro elemento importante en el proceso de profesionalización de la tríada en el campo de la medicina tropical fueron las instituciones y servicios públicos como la ESP-MG y los institutos. Durante su estancia en los diferentes institutos, las mujeres «lo hicieron todo», ocupándose de las más variadas actividades de producción técnica e intelectual.
